# Standard radiotherapy but not chemotherapy impairs systemic immunity in non-small cell lung cancer

**DOI:** 10.1080/2162402X.2016.1255393

**Published:** 2016-11-08

**Authors:** Mehrdad Talebian Yazdi, Mink S. Schinkelshoek, Nikki M. Loof, Christian Taube, Pieter S. Hiemstra, Marij J. P. Welters, Sjoerd H. van der Burg

**Affiliations:** a Department of Pulmonology, Leiden University Medical Center, Leiden, the Netherlands; b Department of Medical Oncology, Leiden University Medical Center, Leiden, the Netherlands

**Keywords:** Chemotherapy, immunity, immunotherapy, myeloid cells, radiotherapy, T cells

## Abstract

**Introduction**: Advanced non-small cell lung cancer (NSCLC) is traditionally treated with platinum-based chemotherapy and radiotherapy. Since immunotherapy holds promise for treating advanced NSCLC, we assessed the systemic effects of the traditional therapies for NSCLC on immune cell composition and function.

**Methods**: 84 pulmonary adenocarcinoma patients, treated either with chemotherapy or radiotherapy, were studied. A prospective study of 23 patients was conducted in which the myeloid and lymphoid cell compartments of peripheral blood were analyzed. Changes in cell populations were validated in a retrospective cohort of 61 adenocarcinoma patients using automated differential counts collected throughout therapy. Furthermore, the functional capacity of circulating T cells and antigen-presenting cells (APC) was studied. Blood samples of healthy individuals were used as controls.

**Results**: In comparison to healthy controls, untreated adenocarcinoma patients display an elevated frequency of myeloid cells coinciding with relative lower frequencies of lymphocytes and dendritic cells. Standard chemotherapy had no overt effects on myeloid and lymphoid cell composition nor on T-cell and APC-function. In contrast, patients treated with radiotherapy displayed a decrease in lymphoid cells and a relative increase in monocytes/macrophages. Importantly, these changes were associated with a reduced APC function and an impaired response of T cells to recall antigens.

**Conclusions**: Platinum-based standard of care chemotherapy for NSCLC has no profound negative effect on the immune cell composition and function. The negative effect of prolonged low-dose radiotherapy on the immune system warrants future studies on the optimal dose and fraction of radiotherapy when combined with immunotherapy.

## Abbreviations

APC: antigen-presenting cell
CTL: cytotoxic T cells
DC: dendritic cell
MLR: mixed lymphocyte reaction
MRM: memory response mix
NSCLC: non-small cell lung cancer
OS: overall survival
PBMC: peripheral blood mononuclear cells
PHA: phytohaemagglutinin
SD: standard deviation
SI: stimulation index
SOP: standard operating procedure

## Introduction

Conventional therapies for advanced non-small cell lung cancer (NSCLC) consist of platinum-based chemotherapy combined with a third-generation cytotoxic drug (stage IV) or concurrent chemoradiotherapy (stage III).^1,2^ The traditional view that these standard of care therapies cause immunosuppression and therefore cannot be combined with new and promising forms of immunotherapy for advanced NSCLC, such as immune checkpoint antibodies targeting the programmed death 1 (PD-1) receptor and its ligand PD-L1,^3^ has been challenged in recent years.^4-7^ Next to their direct cytotoxic effect, platinum-based compounds can also promote anticancer immune responses, for example, by enhancing T-cell activation by dendritic cells (DCs) as part of immunogenic cell death. Furthermore, platinum chemotherapy manipulates the tumor microenvironment by sensitizing tumor cells to cytotoxic T cell (CTL)-mediated attack or by upregulation of MHC class I.^8,9^ Radiotherapy has various immunomodulatory effects such as promoting DC maturation,^10^ local production of type-I interferon^11^ as well as upregulation of MHC class I and tumor-specific antigens,^12^ all of which support tumor-specific effector T cells. These insights on the immune-potentiating effects of both chemotherapy and radiotherapy have led to the rationale that combining these conventional anticancer therapies with new forms of T cell-based immunotherapies can have synergistic effects and, possibly, result in improved clinical outcomes.^4-7^

However, the beneficial effects of chemotherapy and radiotherapy on antitumor immunity are mainly documented in animal models and *in vitro* studies. Information on how these therapies affect the human immune system is lacking, in particular, in NSCLC patients. To address this, patients with advanced pulmonary adenocarcinoma, the most prevalent subtype of NSCLC,^13^ treated with platinum-based chemotherapy or chemoradiotherapy were analyzed for the effect of therapy on myeloid and lymphoid cell composition in the circulation. Furthermore, the functionality of circulating T cells and antigen-presenting cells (APC) was investigated upon standard of care therapy. Our study reveals that, while standard platinum-based chemotherapy does not affect immune cell composition and function, radiotherapy has a negative effect on the number and function of circulating T cells and APCs.

## Materials and methods

### Patients and tissue collection

Between March 2011 and April 2014, a prospective study was conducted in patients with histologically proven pulmonary adenocarcinoma, who were treated at the outpatient clinic of the Department of Pulmonology from three hospitals (Leiden University Medical Center, Alrijne Ziekenhuis and Groene Hart Ziekenhuis). Patients were grouped according to their received therapy. Patients in the first group (carboplatin-vinorelbine) were treated with a 21-d chemotherapy cycle in which they were administered carboplatin (AUC 5 regimen, depending on renal function) and vinorelbine (25 mg/m^2^) on day 1, as well as vinorelbine (25 mg/m^2^) monotherapy on day 8. The second group (carboplatin-pemetrexed) was composed of patients treated with carboplatin (AUC 5 regimen) and pemetrexed (500 mg/m^2^) on day 1. In both groups, at least three cycles of chemotherapy were administered every 3 weeks. Patients in the last group (radiotherapy) received a 5-week cycle of concurrent chemoradiotherapy in which a daily radiation dose of 2.75 Gy (delivered in 24 fractions) was preceded by low-dose cisplatin (6 mg/m^2^) as a radiosensitizer. From all patients, venous blood samples were collected prior to treatment with chemotherapy or radiotherapy (baseline) and at least 14 d after cessation of therapy (post therapy). From all blood samples, peripheral blood mononuclear cells (PBMC) were isolated by Ficoll-density centrifugation according to standard operating procedures (SOP)^14^ and used for analysis of recall antigen T-cell response, mixed lymphocyte reaction (MLR) and flow-cytometric phenotyping.

A retrospective cohort of pulmonary adenocarcinoma patients, treated between 2008 and 2014 with at least three chemotherapy cycles of 21 d (carboplatin-vinorelbine and carboplatin-pemetrexed), was analyzed by collecting automated differential counts from blood samples before start of therapy (baseline), at two time points during the 21-d chemotherapy cycle at week 2 and week 3, and at least 14 d after cessation of therapy (post therapy). From patients treated with a 5-week cycle of radiotherapy, we retrieved automated differential counts at baseline, after each week during the 5-week cycle and at least 14 d after radiotherapy was completed (post therapy). Based on the analysis of this historical cohort, we included an additional five adenocarcinoma patients from whom blood samples were taken at baseline, at week 2 and week 3 of the 21-d chemotherapy cycle, for phenotyping and functional analyses.

### Analysis of recall antigen T-cell response

The capacity of T cells to proliferate upon stimulation with recall antigens was assessed in a 3-d proliferation assay with memory response mix (MRM), composed of tetanus toxoid (Netherlands Vaccine Institute), tuberculin purified protein derivative (Netherlands Vaccine Institute), and Candida (HAL Allergenen Lab), as previously published by our research group.^15^ Briefly, cryopreserved PBMCs were thawed and in triplicate wells (1.0 × 10^5^ cells/well) exposed to three conditions: medium control (90% IMDM supplemented with 10% human AB serum (PAA laboratories, Pasching, Austria)), MRM and Phytohaemagglutinin (PHA, 0.5 µg/mL), which was used as a positive control. Proliferation was measured by ^3^H-thymidine incorporation during the last 18 h of the assay. A positive response was defined as equal or above a stimulation index (SI) of 3. This SI was calculated by dividing the mean counts of stimulated wells by that of the medium control wells.

### Mixed lymphocyte reaction (MLR)

To assess the antigen-presenting capacity of patient PBMCs, a MLR was performed according to SOP.^15^ Thawed patient PBMCs were irradiated at 3,000 rad to prevent proliferation of cells. Next, 1 × 10^5^ patient cells/well were seeded in medium in four replicate wells and co-cultured for 7 d with HLA-mismatched third party non-irradiated lymphocytes (1 × 10^5^ cells/well) isolated from buffy coats obtained from healthy donors. Proliferation was measured by ^3^H-thymidine incorporation during the last 18 h of the assay. As negative controls, irradiated patient PBMCs alone and third party non-irradiated PBMCs alone were used. A positive response was defined as a SI index of at least 3, which was calculated as the mean counts in wells containing irradiated patient PBMCs co-cultured with third party lymphocytes divided by the mean counts of unstimulated third party lymphocytes.

### Flow-cytometric phenotyping of PBMCs

Flow cytometry was used to assess the composition of patient PBMCs before, during and after treatment. PBMCs of eight healthy donors were analyzed for comparison. PBMCs were thawed, washed and stained according to SOPs^14,16^ for the T-cell markers by using antibodies against CD3 (Pacific Blue; DAKO, Glostrup, Denmark), CD4^+^ (PE-CF594; BD Biosciences, San Jose, CA, USA), CD8^+^ (APC-Cy7; BD) and inhibitory T cell markers PD-1 (BV-605, BioLegend, San Diego, CA, USA), NKG2A (AF700, BD), TIM3 (PE, BioLegend) and CTLA-4 (PE-Cy7; BD). Furthermore, in parallel PBMCs were stained with a set of monocyte/macrophage and DC markers. This set was comprised of antibodies against CD1a (FITC; BD), CD3 (Pacific Blue; DAKO), CD11b (PE, BD), CD11c (Alexa Fluor 700; BD), CD14 (PE-Cy7; BD), CD16 (PE-CF594; BD), CD19 (Brilliant Violet 605; BD), CD45 (PerCP-Cy5.5; BD), CD163 (APC; R&D Systems, Minneapolis, MN, USA), CD206 (APC-Cy7; BioLegend) and HLA-DR V500 (BD;). Finally, in a separate experiment PBMCs were stained for regulatory T cell (Treg) markers CD3 (V500, BD), CD4^+^ (AF700, BD), CD25 (PE-Cy7, BD), CD127 (BV650, BD), Foxp3 (PE-CF594, BD), Ki67 (FITC, eBiosciences), Helios (APC, Biolegend) and CD45RA (APC-H7, BD). For each sample, 1,000,000 events were acquired by flow cytometry (BD LSR Fortessa). Data analysis was performed with BD FACSDiva software (version 6.2) using a gating strategy that was recently published by our research group.^15,16^ This strategy is shown in Fig. S1.

Tregs were classified into three subtypes according to a recent paper by Santegoets et al.^17^: Def 1: CD4^+^ CD25^+^ CD127low Foxp3^+^; Def 1 (activated Tregs): CD4^+^ CD25^+^ CD127low Foxp3^+^Ki67^+^; Def 2: CD4^+^ CD25^+^ CD127low Foxp3^+^ Helios^+^; Def 3a: Foxp3high CD45RA^−^ (activated Treg); Def 3b: Foxp3int CD45RA^+^ (naïve Treg). Myeloid and lymphoid cells were defined using the myeloid and lymphoid cell gate within the CD45^+^ gate. Monocytes/macrophages were defined as CD45^+^ HLA-DR^+^ CD1a^−^ CD14^+^ CD11b^+^, with subsets of M1 (CD206^−^CD163^−^), M2a (CD206^+^CD163^−^) and M2c (CD206^−^CD163^+^) monocytes/macrophages. DCs were defined as CD45^+^ HLA-DR^+^ CD1a^−^ CD11b^−^CD14^−^CD11c^+^CD206^−^CD163. Myeloid-derived suppressor cells (MDSCs) were characterized in the myeloid cell population according to recent literature describing 10 separate MDSC subtypes.^18^

### Statistical analysis

The Mann–Whitney *U* test, a non-parametric test for independent samples, was used for statistical comparison of untreated pulmonary adenocarcinoma patients and healthy control PBMCs with regards to lymphoid and myeloid cell populations. For the patients who underwent chemotherapy or radiotherapy, the Wilcoxon signed-rank test (non-parametric test for paired samples) was used to compare recall antigen T-cell response and MLR at baseline and post therapy. This test was also used for analysis of T cell, monocyte/macrophage and DC markers throughout therapy. Percentages within the CD45^+^ gate (monocytes/macrophage and DC markers) or the lymphoid gate (T cell markers) were used in non-parametric testing. A *p*-value < 0.05 was considered statistically significant. Statistical software package SPSS 20.0 (SPSS, Chicago, IL, USA) was used for data analysis.

### Ethical approval

The study protocol was submitted to and approved by the Medical Ethical Committee of the Leiden University Medical Center (P10.187). All patients gave written informed consent prior to participation.

## Results

Twenty-three pulmonary adenocarcinoma patients (14 males, 9 females) with a median age of 61 y (range 48–79 y) were prospectively analyzed. Ten patients were treated with carboplatin-vinorelbine, seven patients received carboplatin-pemetrexed treatment and six patients underwent radiotherapy. The group of eight healthy controls, who supplied donor PBMCs consisted of seven females and one male with a median age of 55 y (range 38–67 y). A summary of patient characteristics is displayed in Table 1.

**Table 1.** Summary of patient characteristics.

Number of patients with pulmonary adenocarcinoma	n = 23
Age, median (range)	61 y (48–79 y)
Gender, Male/Female	14/9
Stage	
I/II	n = 2
III	n = 11
IV	n = 10
Treatment	
Carboplatin-vinorelbine	n = 10
Carboplatin-pemetrexed	n = 7
Radiotherapy	n = 6

### Pulmonary adenocarcinoma patients display cancer-driven changes in lymphoid and myeloid cell frequency

**Figure 1.**
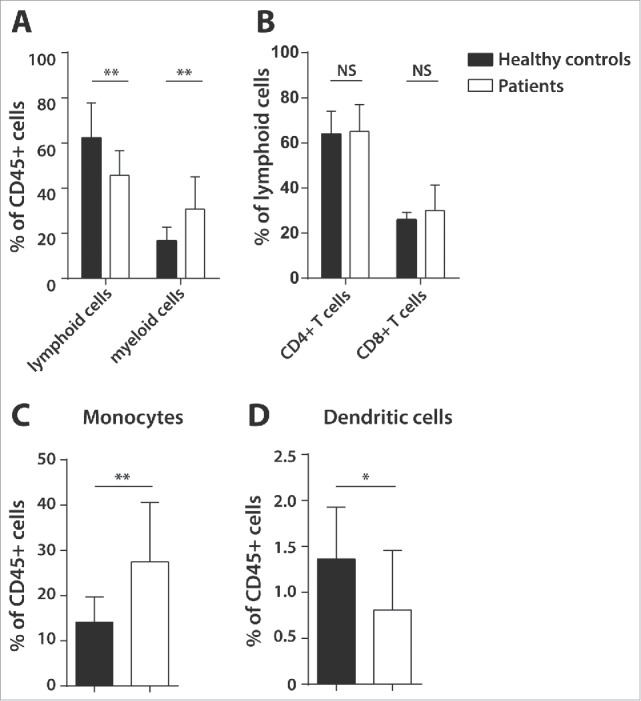
Baseline differences in lymphoid and myeloid cells in healthy controls and pulmonary adenocarcinoma patients. Flow-cytometric phenotyping of peripheral blood mononuclear cells (PBMCs) of 23 untreated pulmonary adenocarcinoma patients and eight healthy donors was performed to analyze myeloid cells, lymphoid cells, CD4^+^ and CD8^+^ T cells, monocytes/macrophages (CD45^+^HLA-DR^+^ CD1a^−^CD11b^+^/CD14^+^) and dendritic cells (CD11b^−^/CD14^−^ CD11c^+^/CD206^−^/CD163^−^) using a gating strategy presented in Supplementary Fig. 1. Shown are lymphoid and myeloid cells (A), CD4^+^ and CD8^+^ T cells as percentage of the lymphoid gate (B), monocytes/macrophages (C) and dendritic cells (D) as percentages of the CD45^+^ gate. Data is shown as mean with standard deviation (SD) and Mann–Whitney *U* test was used for statistical analysis (**p < 0.01, *p < 0.05, NS = non-significant).

First, baseline differences in circulating myeloid and lymphoid cell populations between untreated pulmonary adenocarcinoma patients and healthy controls were analyzed (Fig. 1). Patients showed a significantly elevated level of myeloid cells coinciding with a lower frequency of circulating lymphoid cells (Fig. 1A) independent of their gender. Although no difference was observed between patients and healthy controls with respect to CD4^+^ and CD8^+^ T cell frequency gated as percentage of the lymphoid gate (Fig. 1B), a lower frequency of CD4^+^ T cells was noted in adenocarcinoma patients when gated as percentage of the CD45^+^ gate, and a similar trend was seen for CD8^+^ T cells, fitting with the observation that the percentage of lymphoid cells is decreased, whereas the percentage of myeloid cells is increased (Fig. S2). Subset analysis of the myeloid cell populations, demonstrated a higher percentage of CD11b^+^/CD14^+^ monocytes/macrophages (Fig. 1C) and a lower percentage of CD11b^−^CD14^−^CD11c^+^CD206^−^CD163^−^ DCs (Fig. 1D) in the PBMC of the patients when compare with that of healthy donors. No difference was observed with respect to MDSC frequency and expression of inhibitory T cell markers between adenocarcinoma patients and healthy donors (Fig. S3). Thus, adenocarcinoma patients show a tumor-associated abnormal level of myeloid cells which is mainly caused by increased numbers of monocytes/macrophages while in parallel the frequency of circulating lymphoid cells and DCs is lower.

### Changes in immune cell composition after standard of care treatment for NSCLC

**Figure 2.**
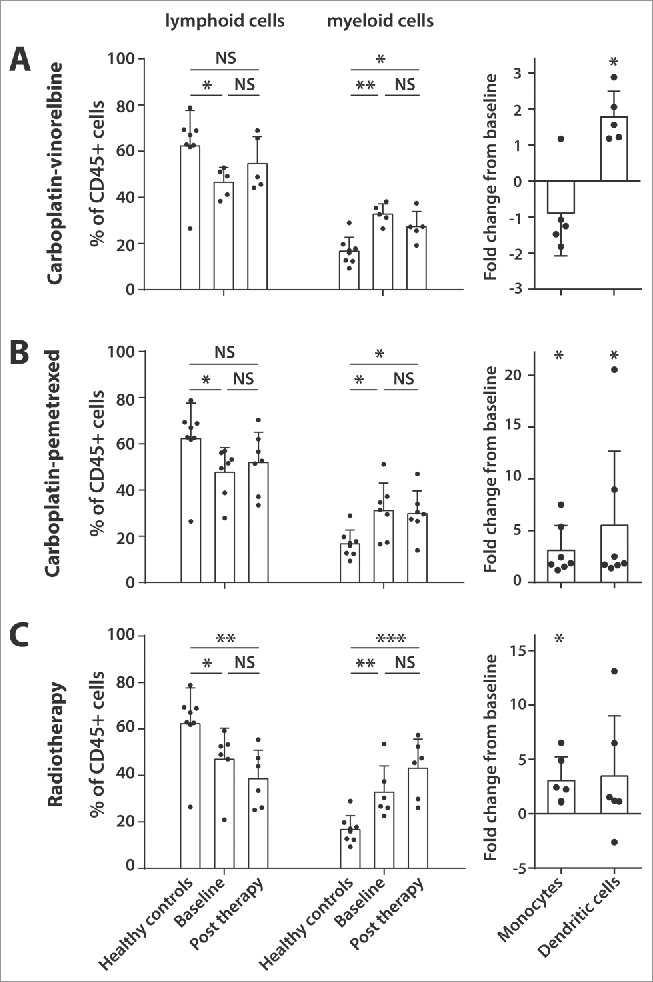
Effect of standard of care therapies for advance pulmonary adenocarcinoma on lymphoid and myeloid cell populations. Peripheral blood of 18 pulmonary adenocarcinoma patients was analyzed for lymphoid and myeloid cell composition (depicted as percentages of the CD45^+^ gate) as well as changes in monocyte/macrophage (CD14^+^ CD11b^+^) and dendritic cell (CD11b^−^CD14^−^CD11c^+^CD206^−^CD163^−^) frequency. This was done prior to treatment (baseline) and at least 14 d after cessation of therapy (post therapy). Three treatment groups were assessed: carboplatin-vinorelbine (A, n = 5), carboplatin-pemetrexed (B, n = 7) and radiotherapy (C, n = 6). Healthy donor PBMC were taken along for comparison. The fold change of post therapy from baseline is given for the patients concerning the monocyte/macrophage and dendritic cell frequencies. Data is shown as mean with SD and Mann–Whitney *U* test (healthy controls compare with patients at baseline) and Wilcoxon signed rank test (patients at baseline compare with post therapy) were used for statistical analysis (***p <0.001, **p < 0.01, *p < 0.05, NS = non-significant).

Next, the effect of standard of care NSCLC treatment modalities on immune cell composition was assessed. In the patient group treated with carboplatin-vinorelbine (n = 5), treatment did not result in overt changes in the percentage of lymphoid and myeloid cells. However, subset analysis of the myeloid cell population showed that carboplatin-vinorelbine treatment caused an increase in DCs and a decrease in monocytes/macrophages (Fig. 2A). Treatment with carboplatin-pemetrexed (n = 7) similarly had no profound effect on the composition of the myeloid and lymphoid cell compartments. Here, a treatment-related increase was observed for both the percentage of monocytes/macrophages and the percentage of DCs (Fig. 2B).

The treatment of patients with radiotherapy (n = 6) resulted in a slight decrease in the percentage of lymphoid cells and a concomitant increase in myeloid cells, which was not likely to be associated with more extensive irradiation as there is no relation between the irradiated tumor volume and extent of lymphopenia in this small group of patients (Fig. S4). The lower frequency of lymphocytes as compared with healthy donors remained after cessation of radiotherapy. An increase of the percentage of monocytes/macrophages was observed (Fig. 2C). All three treatment groups did not display changes in the frequency of T cells expressing inhibitory markers (Fig. S3) or Tregs (Fig. S5). Patients treated with radiotherapy displayed a significant increase in the frequency of MDSC type 4 (CD14^+^ HLA-DR^low^) and MDSC type 7 (CD14^+^ CD33^+^ HLA-DR^low^) cells (Fig. S3).

### Standard of care NSCLC therapies cause transient and permanent changes in absolute lymphocyte and monocyte counts during therapy

**Figure 3.**
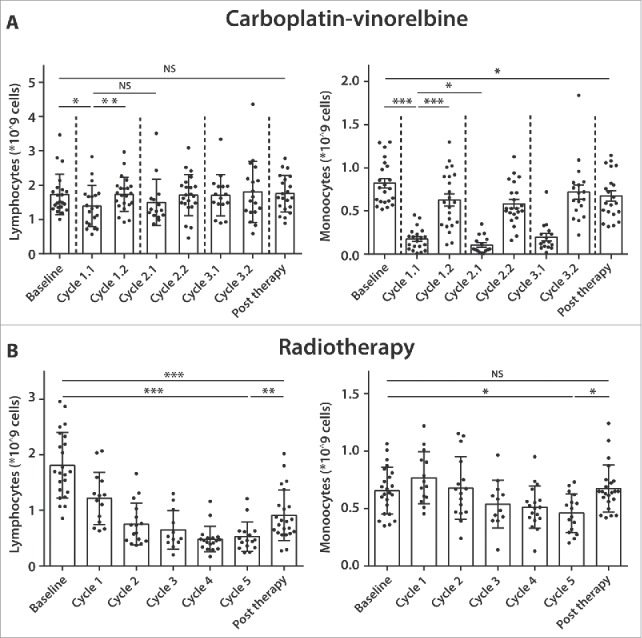
Standard of care therapies for pulmonary adenocarcinoma cause transient and permanent changes in absolute lymphocyte and monocytes counts during therapy. From 22 pulmonary adenocarcinoma patients treated with at least three cycles of carboplatin-vinorelbine (A), automated leucocyte differential counts were retrospectively collected at two time points (at week 2 and week 3) during a 21-d cycle of chemotherapy. Weekly automated leucocyte differential counts were also collected from 23 patients treated with a 5-week cycle of radiotherapy (B). From all patients, counts were retrieved at baseline and post therapy. Results are presented as mean lymphocyte and monocyte counts (per 10^9^ cells) with SD. Wilcoxon signed rank test was used for statistical analysis (***p <0.001, **p < 0.01, *p < 0.05, NS = non-significant).

To validate these observations and to obtain more detailed insight into the changes in immune cell composition during treatment, we analyzed the lymphocyte and monocyte counts in a historical cohort of pulmonary adenocarcinoma patients for whom automated leucocyte differential counts were retrieved (Fig. 3). In 22 patients, who were treated with carboplatin-vinorelbine (Fig. 3A), a small drop in absolute lymphocyte cell counts from baseline (mean 1.87 × 10^9^ cells) was observed in week 2 of the first cycle (cycle 1.1, mean 1.41 × 10^9^ cells, *p* <0.05), which normalized at week 3 within the same cycle (cycle 1.2, mean 1.63 × 10^9^ cells, *p* <0.01). Lymphocyte counts remained stable during therapy, and there was no effect on absolute lymphocytes counts post therapy when compare with baseline (mean 1.61 × 10^9^ cells, *p* = 0.98). On the other hand, carboplatin-vinorelbine treatment caused a strong decline in absolute monocyte counts from baseline (mean 0.81 × 10^9^ cells), which is evident in week 2 of the first cycle (cycle 1.1, mean 0.22 × 10^9^ cells, *p* <0.001), but almost recovers a week later during the same 21-d cycle (cycle 1.2, mean 0.57 × 10^9^ cells, *p* <0.001), only to drop again sharply in the next chemotherapy cycle (cycle 2.1, mean 0.09 × 10^9^ cells, *p* < 0.05). After therapy cessation, absolute monocyte cell counts show a quick recovery nearly to the baseline level (mean 0.75 × 10^9^ cells, *p* < 0.05), explaining the results of our prospective patient group (Fig. 2A). The analysis of 16 patients treated with carboplatin-pemetrexed showed no changes in absolute numbers of lymphocytes and monocytes during or after the course of chemotherapy (Fig. S6).

Finally, 23 adenocarcinoma patients treated with radiotherapy were analyzed (Fig. 3B). The baseline absolute lymphocyte numbers (mean 1.88 × 10^9^ cells) showed a significant decline during the course of radiotherapy, which persisted in the last week of the 5-week treatment cycle (Cycle 5, mean 0.49 × 10^9^ cells, *p* < 0.001) and despite a recovery after completion of treatment, the total lymphocyte counts remained lower than baseline (post therapy, mean 0.96 × 10^9^ cells, *p* <0.01). Baseline monocyte counts (mean 0.68 × 10^9^ cells) generally remained stable during radiotherapy treatment, with only a small drop observed after week 5 (Cycle 5, mean 0.44 × 10^9^ cells, *p* < 0.05). This modest effect disappeared after treatment (post therapy, mean 0.67 × 10^9^ cells, *p* = 0.90). The absolute loss in lymphocytes and stable number of monocytes explains the treatment-related relative drop in the lymphocyte to monocyte ratio observed in our prospective patient group (Fig. 2C).

### Radiotherapy impairs recall T-cell responses and APC function in patients with pulmonary adenocarcinoma

**Figure 4.**
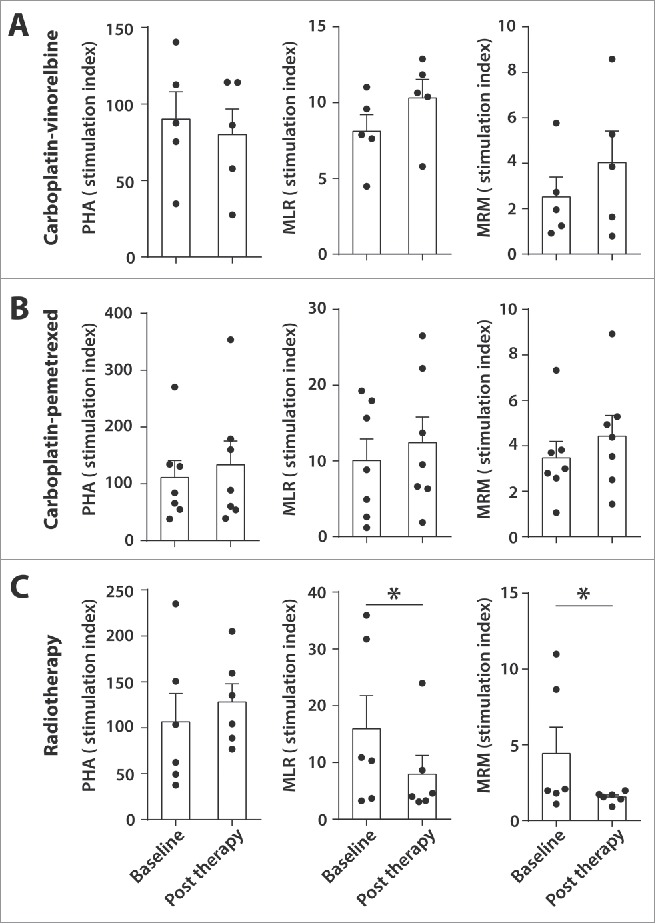
Effect of standard of care pulmonary adenocarcinoma therapies on T cell and APC function PBMCs of 18 patients with pulmonary adenocarcinoma were tested for the capacity of T cells to proliferate upon stimulation with phytohaemagglutinin (PHA) or with recall antigens (memory response mix, MRM). Antigen-presenting cell (APC) function was tested by mixed leucocyte reaction (MLR). Three treatment groups were investigated: carboplatin-vinorelbine (A, n = 5), carboplatin-pemetrexed (B, n = 7) and radiotherapy (C, n = 6). Data is shown as mean stimulation index (SI) at baseline and at least 14 d after cessation of therapy (post therapy). Wilcoxon signed rank test was used for statistical analysis (***p < 0.001, **p < 0.01, *p < 0.05, NS = non-significant).

Subsequently, we tested the impact of these therapies on the stimulatory capacity of APCs and on the responsiveness of T cells to antigenic stimulation (Fig. 4). The APCs isolated from patients, who were treated with either carboplatin-vinorelbine or carboplatin-pemetrexed, displayed a good capacity to stimulate allogeneic lymphocytes both at baseline and after chemotherapy in a MLR assay. Moreover, none of the chemotherapy schedules influenced the T-cell response to a mix of recall antigens (MRM). In addition, T-cell reactivity to mitogenic stimulation with PHA was strong at baseline and not affected by chemotherapy (Figs. 4A and B). Based on the marked decrease in absolute monocyte counts within the 21-d carboplatin-vinorelbine treatment (Fig. 3A), we prospectively collected PBMCs from five additional patients at three time points (baseline, week 2 and week 3) during the course of their chemotherapy cycle (Fig. S7). In these five patients, the absolute decrease in monocytes observed in their leucocyte differential counts (Fig. S7A) was reflected by a relative decrease in myeloid cells (*p* < 0.05) and a concomitant increase in lymphoid cells (*p* = 0.06) (Fig. S7C). The sharp decline in myeloid cells was mirrored by a drop in monocytes/macrophages. Analysis of M1/M2a/M2c monocytes/macrophages showed a similar decline in all subsets (Fig. S7C). The observed changes in myeloid cells did not lead to overt changes in the response of T cells to recall antigens or that of APC to stimulate allogeneic T cell reactivity (Fig. S7B).

In contrast, the T cells of patients, who were treated with radiotherapy, demonstrated a significant decline in their reactivity to recall antigens (Fig. 4C, mean baseline MRM response SI index 4.4, post therapy mean MRM response 1.5, *p* < 0.05). This decline in T-cell reactivity coincided with a weaker MLR response post radiotherapy (mean SI index 7.9) compare with baseline (mean SI index 16.0, *p* < 0.05). Since the PHA-stimulated T cells showed a good proliferative response before (mean SI index 106.4) and after radiotherapy (mean SI index 128.2), reflecting an intact intrinsic capacity of T cells to proliferate, our data suggest that the negative effect of radiotherapy upon the proliferative capacity of PBMCs to stimulation with recall antigens might be related to a hampered APC function.

Taken together, standard of care chemotherapy schedules for pulmonary adenocarcinoma have no profound effect on both the levels and function of myeloid and lymphoid cells in the peripheral blood. The negative effect of radiotherapy on the recall T-cell response can not only be explained by the absolute decrease in lymphoid cells but is also related to the impaired APC function which might be fostered by the relative increase in monocytes/macrophages.

## Discussion

In the present study, we demonstrate that treatment of advanced pulmonary adenocarcinoma patients with radiotherapy has several negative effects on circulating immune cells. The most striking observation is a persistent drop in lymphocyte counts during treatment, which coincides with a decline in reactivity of T cells to recall antigens after completion of radiotherapy. Previously, lymphocytopenia has been observed as a side-effect of radiotherapy^19,20^ and a negative association of lymphocytopenia with OS has been reported, both for NSCLC and other cancer types.^21,22^ Spearman correlation analysis failed to show a relation between the tumor volume irradiated and the observed lymphocytopenia (Fig. S4), however, this might be due to a small sample size (n = 6). The fact that radiotherapy aimed at smaller target volumes can reduce lymphocytopenia in cancer patients is well known and discussed in more detail elsewhere.^23^

Whether radiotherapy also affects lymphocyte function has been less well described in cancer patients. Radiotherapy has been shown to negatively affect T-cell responses to recall antigens in testis carcinoma patients who received adjuvant localized radiotherapy^24^ and in patients with Hodgkin's disease treated with mantle field irradiation.^25^ This study is the first to report a negative functional effect of radiotherapy with respect to circulating T cells in pulmonary adenocarcinoma patients. This effect is unlikely to be caused by infusion with low-dose cisplatin (6 mg/m^2^) that preceded radiotherapy fractions, since cisplatin has been shown to stimulate antitumor immunity, in particular by the induction of tumor-specific CD8^+^ T cells, by promoting infiltration of inflammatory APCs harboring T-cell co-stimulatory ligands into the tumor, and by improving cure rates when combined with long peptide vaccines.^26,27^ The detrimental effect on APC function by radiotherapy before and after therapy (Fig. 4) could in part explain this decline in recall-directed T-cell function. Studies on the association of radiotherapy with antigen-presenting capacity of PBMC in NSCLC patients are scarce. Only one study has reported a decline in MLR response and a concomitant decline in PHA-stimulated T-cell proliferation in NSCLC patients who underwent radiotherapy.^28^ Our finding that radiotherapy hampers APC stimulatory capacity, potentially explained by decreased APC function or by the suppressive effect of MDSC subtypes which are present at higher frequencies after radiation, contradicts data from mouse studies reporting a beneficial role of radiotherapy with respect to DC maturation and activation.^4,6^ For example, one study in C57BL/6 mice bearing B16gp melanoma tumors demonstrated that local irradiation with a single high dose of 10 Gy resulted in upregulation of CD70 and CD86 on local DCs, both of which are co-stimulatory molecules involved in T-cell priming.^10^ In contrast, NSCLC patients in our study received a daily dose of 2.75 Gy with a total of 24 fractions, amounting to a total dose of 66 Gy during the course of 5 weeks. One possible explanation for the discrepancy between our results and findings from mouse studies is that a single high dose or a few fractions of high-dose radiotherapy are needed to recruit circulating immune cells to the tumor site, whereas continuous low dose irradiation over the course of 5 weeks may finally kill these tumor-infiltrating cells before they can even exert their tumoricidal function. In this light, future studies should address how the immune system is influenced by stereotactic ablative body radiotherapy, a form of high precision radiotherapy with high radiation doses in a few fractions which has achieved excellent tumor control rates in early stage NSCLC.^29^ Regardless, our results indicate that prolonged low-dose radiotherapy negatively affects T-cell and APC function in pulmonary adenocarcinoma patients, a matter which needs to be addressed when considering combinatorial approaches with T-cell-based immunotherapies.

Another important observation from this study is the strikingly high frequency of myeloid cells in the peripheral blood of pulmonary adenocarcinoma compare with healthy subjects (Fig. 1). Elevated levels of circulating myeloid-derived suppressor cells (MDSC) have been previously reported in advanced NSCLC and their presence was negatively associated with the frequency of CD8^+^ T lymphocytes,^30,31^ which is in line with our observation of decreased lymphoid cells in the PBMC of advanced pulmonary adenocarcinoma patients compare with healthy donors. Recently, our research group demonstrated that abnormal levels of circulating myeloid cells in cervical carcinoma patients were shown to normalize after treatment with carboplatin and paclitaxel. Importantly, this was associated with higher T-cell reactivity against common microbial recall antigens.^15^ In the present study, advanced pulmonary adenocarcinoma patients, who were treated with carboplatin and vinorelbine also demonstrated a sharp decline in circulating myeloid cell populations (Fig. 3), but this did not result in changes in recall antigen response to MRM or changes in ability of APCs to stimulate allogeneic T-cell proliferation (MLR response) (Fig. S7). Several factors might explain this apparent discrepancy between our pulmonary adenocarcinoma patients and the cervical cancer patients, such as the use of a different third generation cytotoxic drug in this study cohort. Another important contributing factor might be the impaired APC function of the adenocarcinoma patients at baseline. This is not only reflected by the relative low frequency of DCs in the treatment-naive patients (Fig. 1), but moreover by the relatively weak MLR response at baseline (Fig. 4 mean MLR SI index of 11.5) when compare with cervical cancer patients (mean MLR SI index of > 50).^15^ This difference in APC function of advanced NSCLC patients might be related to smoking, which is the main risk factor for development of NSCLC.^13^ *In vitro* studies have shown that nicotine diminishes DC activation and maturation thereby promoting T-cell polarization to the T-helper 2 phenotype.^32,33^ Nevertheless, the effect of carboplatin-vinorelbine therapy on reducing levels of immunosuppressive myeloid cell populations in pulmonary adenocarcinoma patients is remarkable and further studies into its effect on antitumor T-cell immunity are warranted.

In conclusion, this study shows that standard platinum-based chemotherapy used in advanced pulmonary adenocarcinoma patients has no profound adverse effects with respect to immune cell composition and function. Hence, no theoretical objection exists to combining these chemotherapy schedules with novel T-cell-based immunotherapies. However, the decline in function of circulating T cells and APCs caused by prolonged low-dose radiotherapy impairs systemic immunity in these patients, which warrants further debate and studies on how radiation can be optimally applied (e.g., by hypofractionated treatment targeting smaller volumes^23^) in order to sustain antitumor immunity and perhaps to act synergistically when combined with new forms of T-cell-based immunotherapy in NSCLC patients.

## Supplementary Material

KONI_A_1255393_s02.docx
