# Supplementary material for: Standard radiotherapy but not chemotherapy impairs systemic immunity in non-small cell lung cancer
Source: Oncoimmunology. 2016 Nov 8;5(12):e1255393. doi: 10.1080/2162402X.2016.1255393 (PMC5214754; doi:10.1080/2162402X.2016.1255393)
Supplement: KONI_A_1255393_s02.docx [file koni-05-12-1255393-s001.docx]

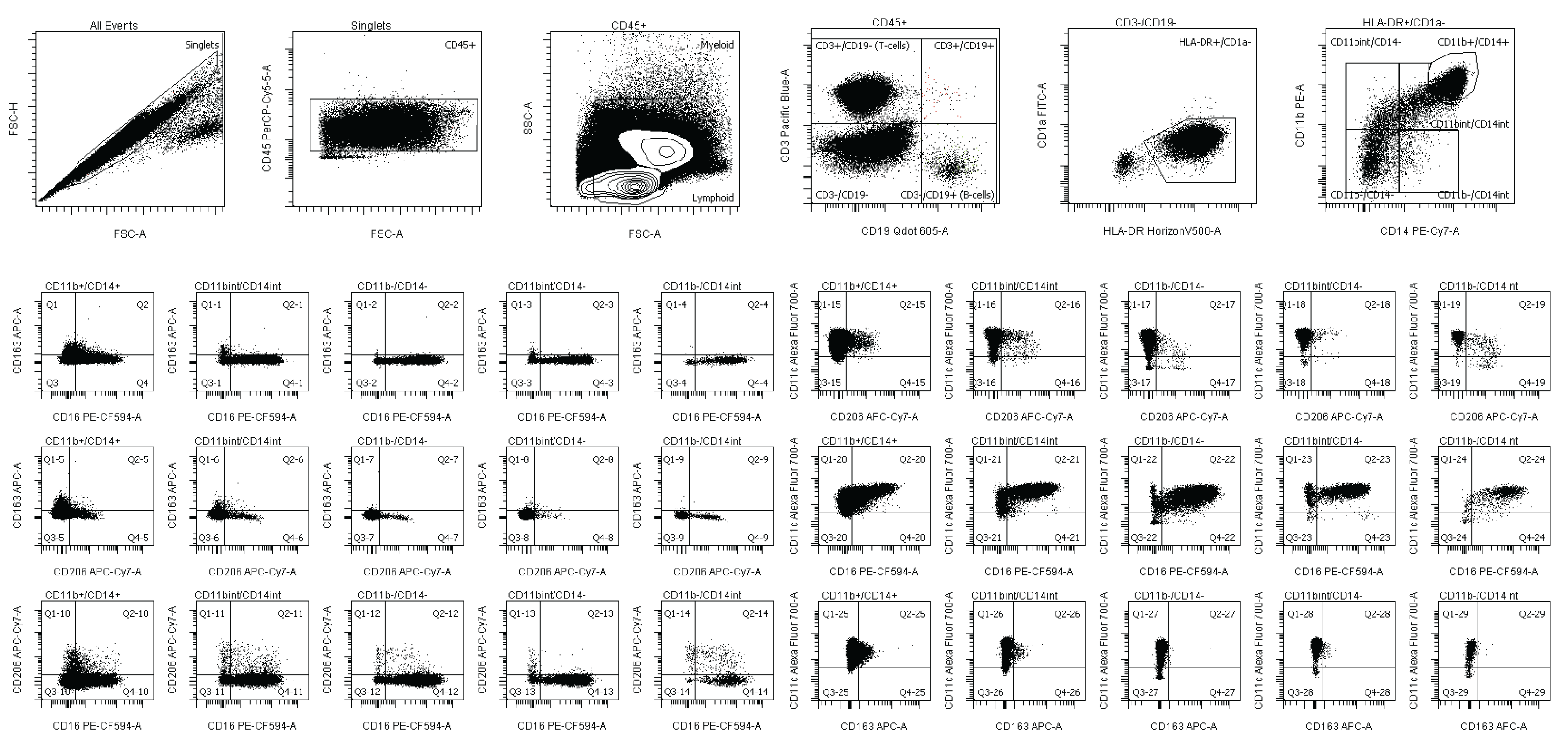


**Supplementary Figure 1. Gating strategy for myeloid and lymphoid cell phenotyping**

Singlets were gated based on FCS-A and FCS-H to exclude dead cells. Then, leucocytes were selected based on high expression of CD45. The CD45+ cells were divided into myeloid cells and lymphoid cells with use of the myeloid cell gate and lymphoid cell gate within the forward-side scatter plots. Also, CD45+ cells were plotted for the expression of CD3 and CD19. T cells (CD3+CD19-) were subdivided in CD4+ and CD8+ T cells. Monocytes/macrophages (CD3-CD19- cells) were gated for HLA-DR+ / CD1a- cells and then plotted for CD14 and CD11b. Based on differential expression of CD163, CD16, CD206 and CD11c, monocytes/macrophages were subdivided into M1/M2a/M2c and DC subsets.

**
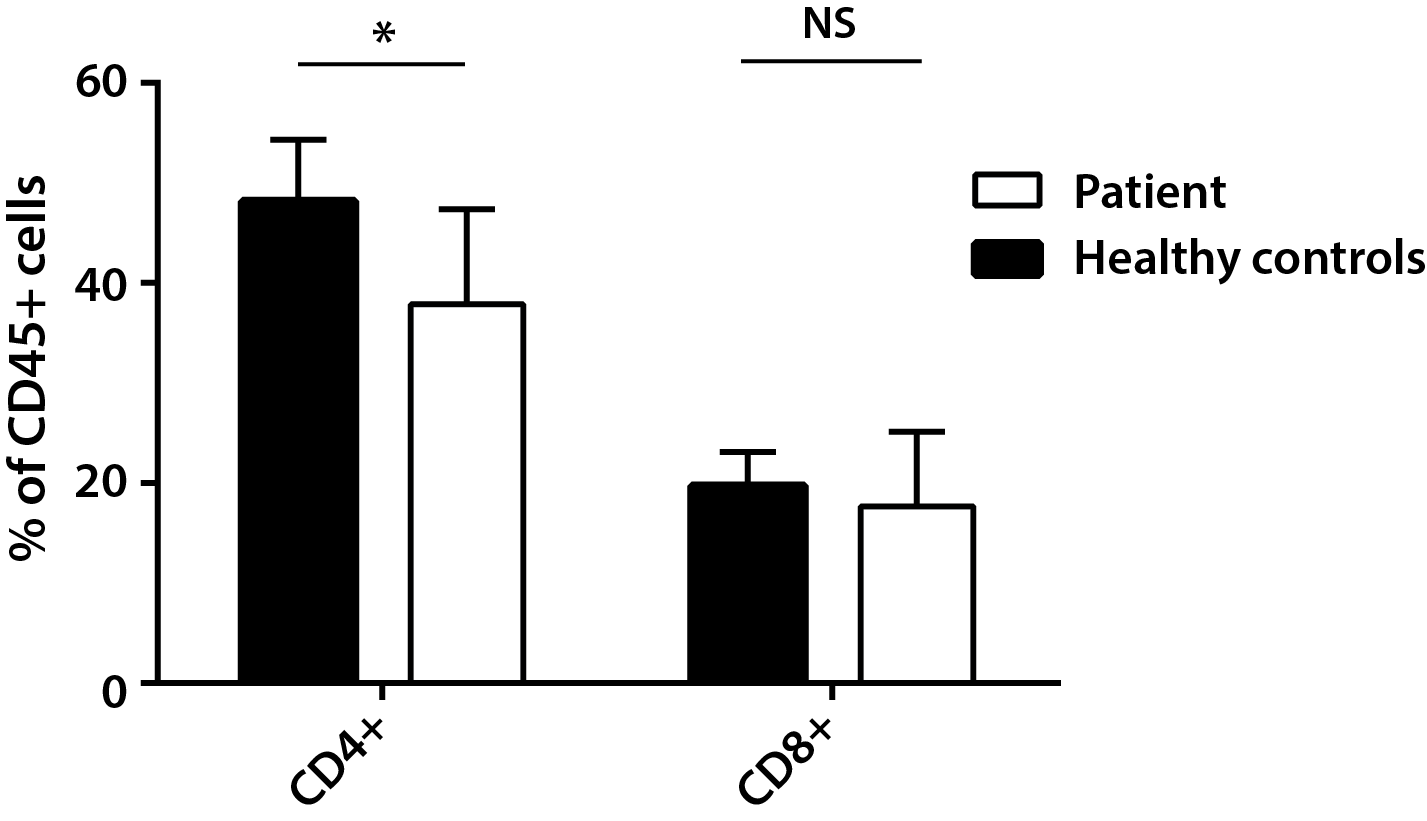
**

**Supplementary Figure 2.**

Flow-cytometric phenotyping of peripheral blood mononuclear cells (PBMCs) of 23 untreated pulmonary adenocarcinoma patients and 8 healthy donors revealed that CD4+ T cells, displayed as percentage of the CD45+ gate, were significantly lower in patients than in healthy controls (p<0.05) whereas the same trend was observed for CD8+ T cells (albeit not significant). Data is shown as mean with SD and statistical analysis was performed using the Mann-Whitney U test (* p< 0.05, NS = non-significant).

**
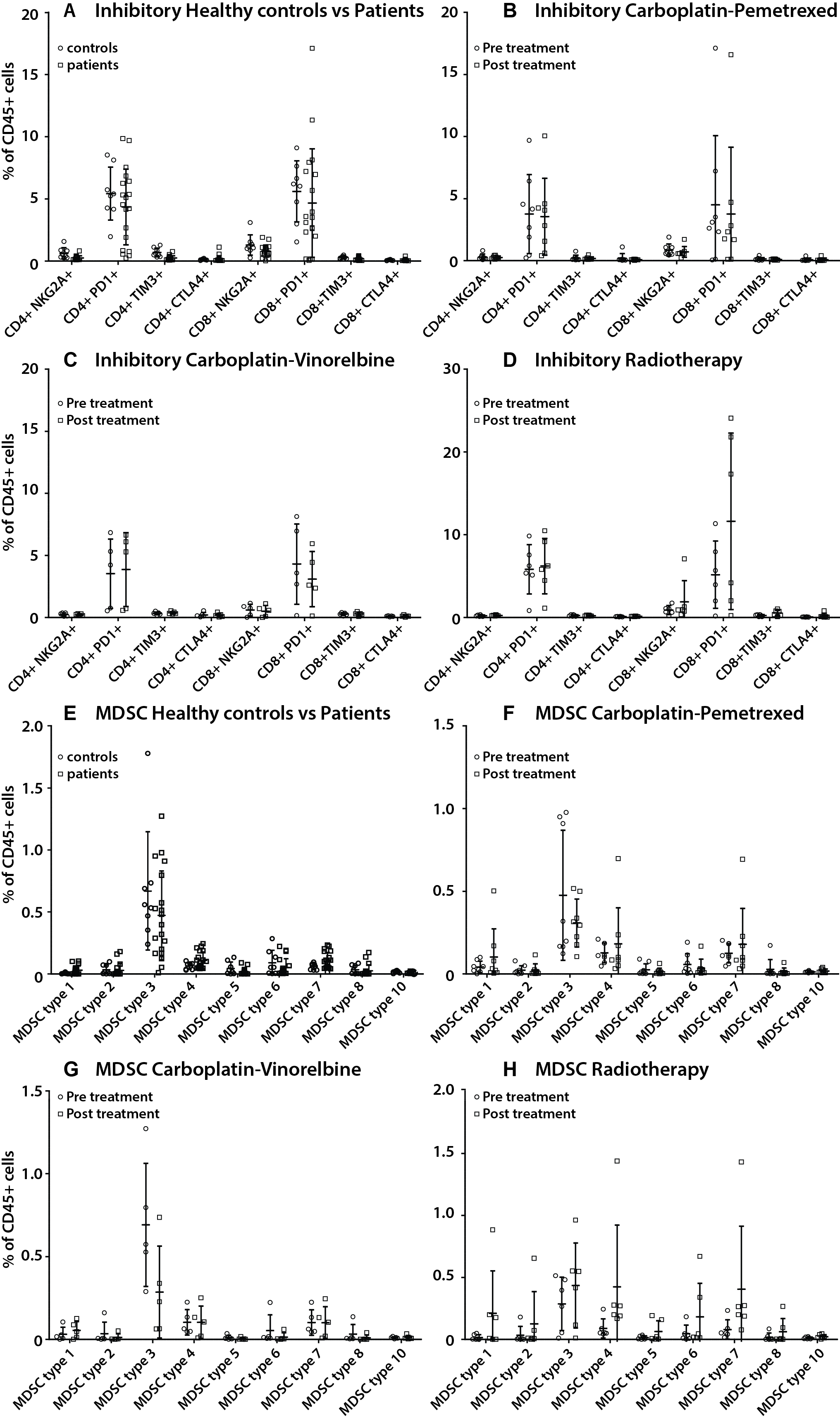
**

**Supplementary Figure 3. Effect of standard of care NSCLC treatment on inhibitory T cell markers and myeloid derived suppressor cells (MDSC).**

PBMCs of pulmonary adenocarcinoma patients were used for analysis of inhibitory T cell markers (PD-1, NKG2A, TIM3 and CTLA-4 on CD4+/CD8+ T cells) and MDSCs at baseline (pre treatment) and at least 14 days after cessation of therapy (post treatment). Healthy donor PBMCs were taken along for comparison. Three treatment groups were assessed: carboplatin-pemetrexed (n=7), carboplatin-vinorelbine (n=5) and radiotherapy (n=6). All three treatments did not induce changes in T cell inhibitory markers (**B-D**, p> 0.05). Comparison of healthy controls and patients at baseline also did not show a difference in these markers (**A**, p>0.05). Treatment with doublet chemotherapy (carboplatin-pemetrexed and carboplatin-vinorelbine) was not associated with changes in MDSCs (**F-G**, p> 0.05), whereas a significant increase in MDSC type 4 (CD14+ HLA-DRlow) and MDSC type 7 (CD14+ CD33+ HLA-DRlow) was observed in patients treated with radiotherapy (**H**, p<0.05). Frequency of MDSCs was comparable between healthy controls and patients at baseline (**E**, p>0.05). Data is shown as mean with SD and Wilcoxon signed rank test (patients at baseline compared to post therapy) and Mann-Whitney U test (healthy controls compared to patients at baseline) were used for statistical analysis.


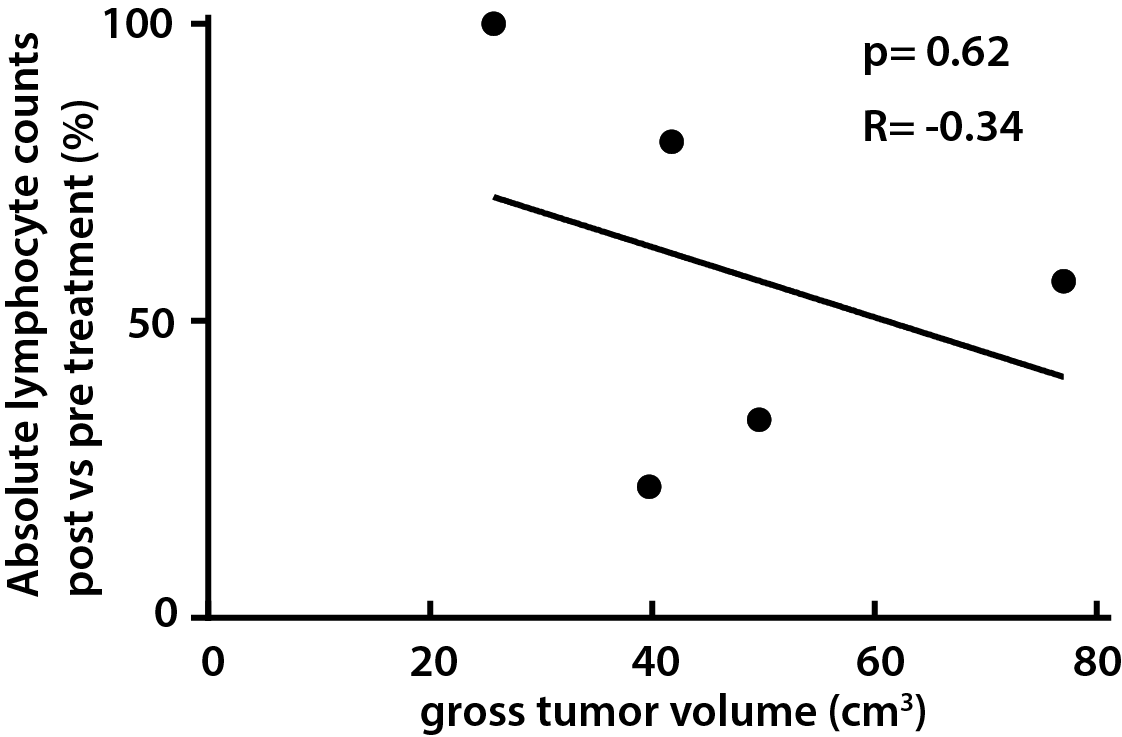


**Supplementary Figure 4. Effect of irradiated tumor volume on extent of lymphopenia**

To investigate to which extent irradiated tumor volume, expressed as gross tumor volume, had an effect on the decrease in lymphocytes in the blood of pulmonary adenocarcinoma patients, lymphocyte counts pre- and post-radiotherapy were collected from five out of six patients who underwent radiotherapy.

Spearman correlation analysis failed to show a relation between the tumor volume irradiated and the observed lymphopenia (R=-0.34, p=0.62).

**
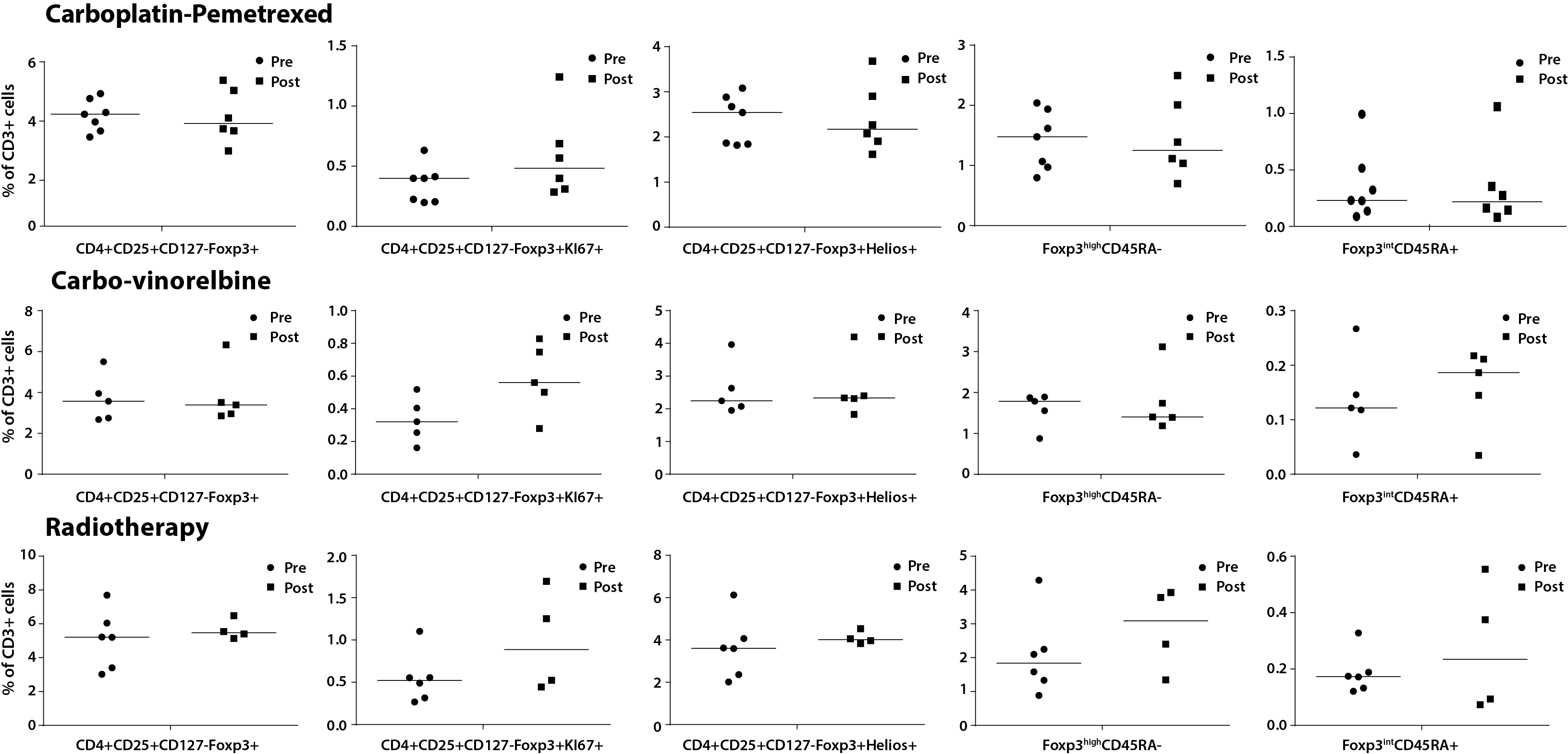
**

**Supplementary Figure 5. Effect of standard of care NSCLC treatment on regulatory T cells (Tregs)**

PBMCs of pulmonary adenocarcinoma patients were used to assess changes in Treg frequency. Three definitions of Tregs were used:

Def. 1: CD4+CD25+CD127^low^Foxp3+ and CD4+CD25+CD127^low^Foxp3+Ki67+ (activated Tregs); Def. 2: CD4+CD25+CD127^low^Foxp3+Helios+

Def. 3a: Foxp3^high^CD45RA- (activated Treg); Def. 3b: Foxp3^int^CD45RA+ (naïve Treg). Three treatment groups were assessed: carboplatin-pemetrexed (n=7), carboplatin-vinorelbine (n=5) and radiotherapy (n=6). All three treatments did not induce changes with respect to the different Treg subtypes (p>0.05). Data is shown as mean with SD and Wilcoxon signed rank test was used for statistical analysis.


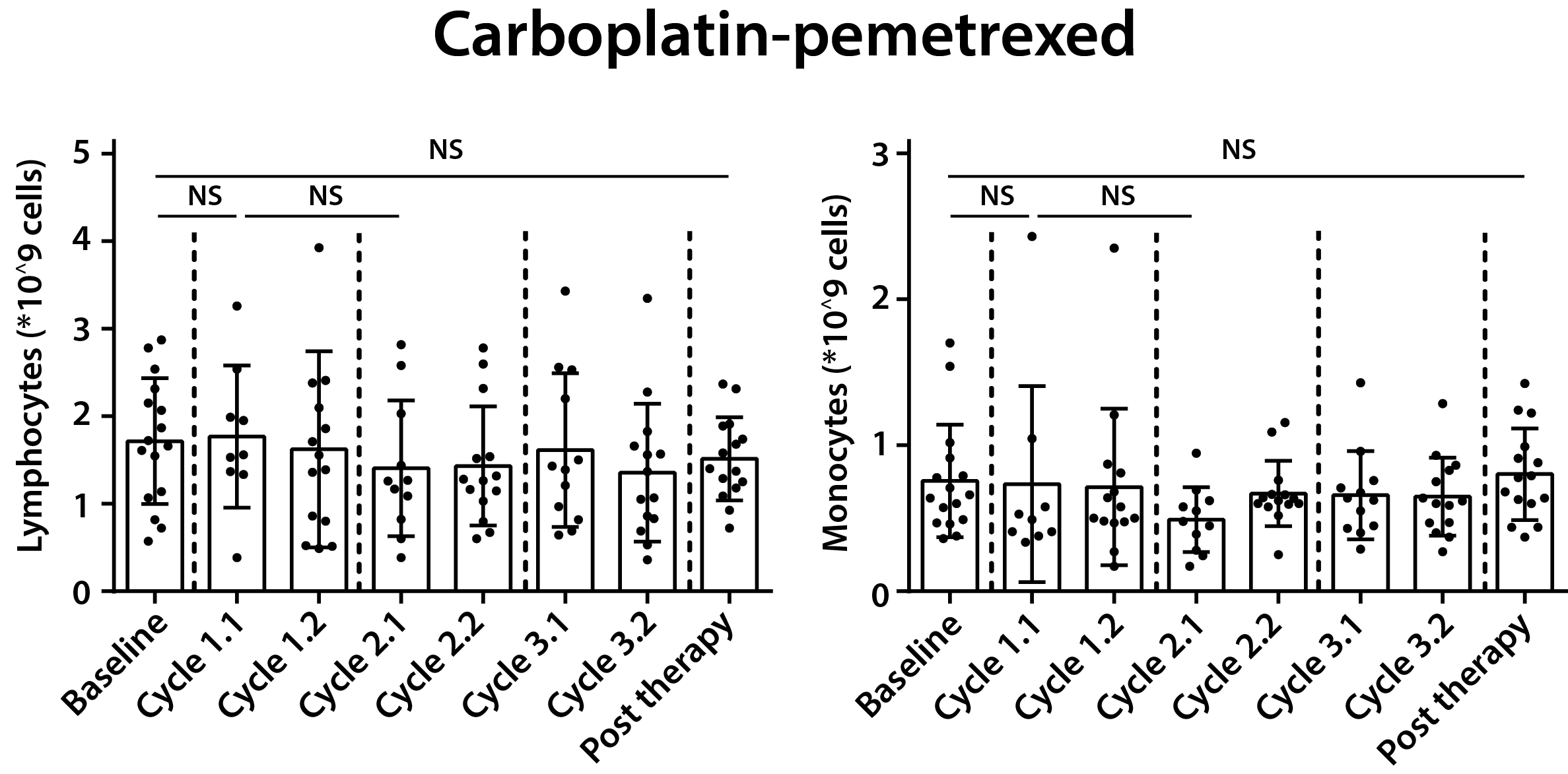


**Supplementary Figure 6. Carboplatin-pemetrexed treatment does not alter absolute lymphocyte and monocytes counts during therapy.**

Automated differential leucocyte counts were retrieved from a historical cohort of 16 pulmonary adenocarcinoma patients treated with at least three cycles of carboplatin-pemetrexed. Automated leucocyte differential counts were collected at baseline, post therapy and at two time points (at week 2 and week 3) during the 21-day cycle of chemotherapy. Results are shown as mean lymphocyte and monocyte counts (per 10^^^9 cells) with SD. Wilcoxon signed rank test was used for statistical analysis (NS = non-significant).


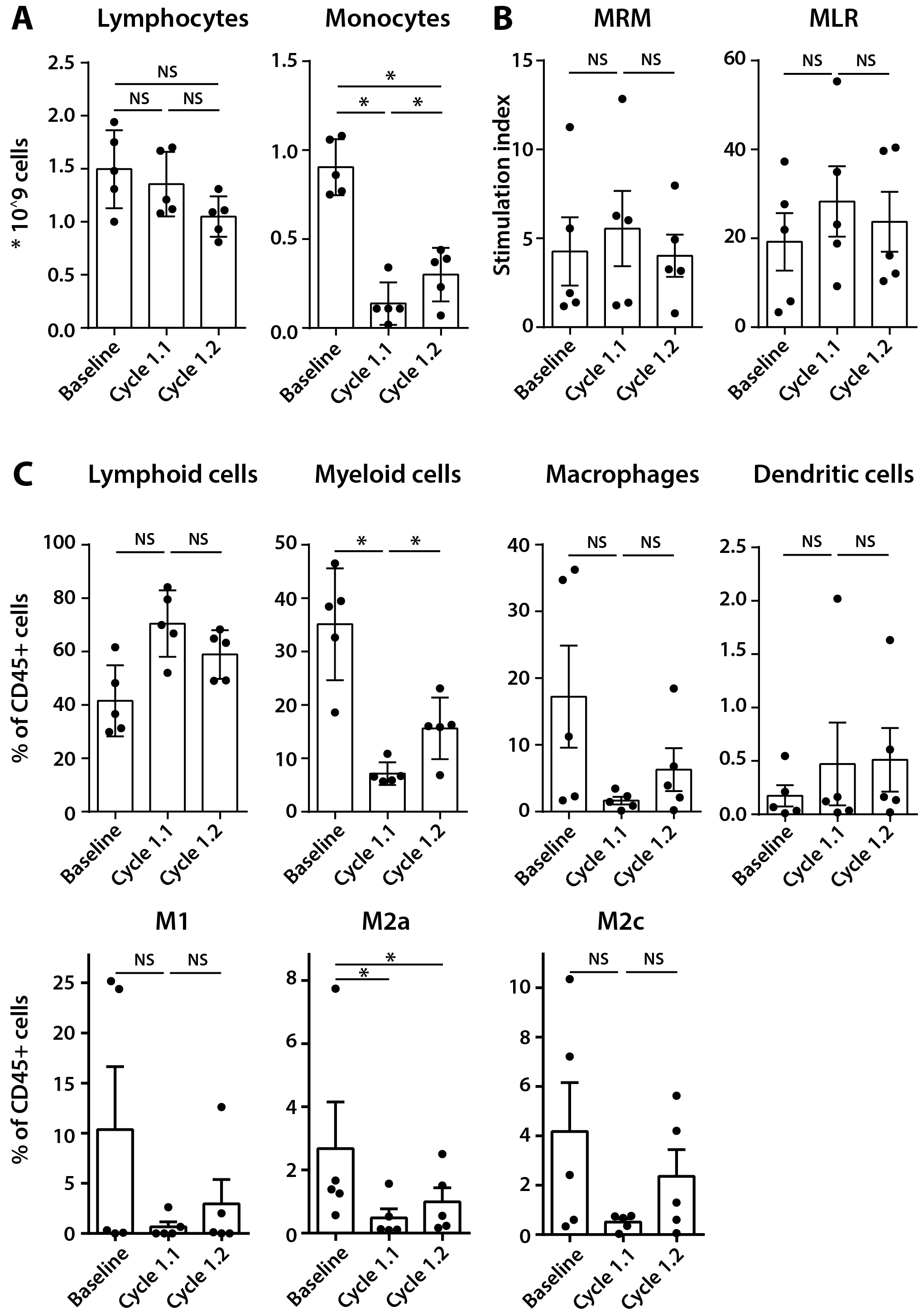


**Supplementary Figure 7. Lymphoid and myeloid cells change during carboplatin-vinorelbine treatment without an effect on T-cell and APC function**

At three time points (baseline, at week 2 and week 3) during a 21-day cycle of carboplatin-vinorelbine, PBMCs of 5 patients were prospectively collected for flow-cytometric phenotyping, testing of recall antigen response (MRM) and ability of APCs to stimulate allogeneic T cell proliferation (MLR). Shown are leucocyte differential counts (per 10^^^9 cells) (**A**), Stimulation index (SI) of MRM and MLR response (**B**), and frequency of lymphoid and myeloid cells, monocytes/macrophages and dendritic cells as percentages of the CD45+ gate. Subset analysis is shown for M1 (CD206-CD163-), M2a (CD206+CD163-) and M2c (CD206-CD163+) monocytes/macrophages (**C**). Results are presented as mean with SD and Wilcoxon signed rank test was used for statistical analysis. (* p< 0.05, NS = non-significant)
